# Unmet health care needs among the working-age population: Evidence from the Great Recession in Spain (2008-2012)

**DOI:** 10.23938/ASSN.1093

**Published:** 2024-12-24

**Authors:** Carla Blázquez-Fernández, David Cantarero-Prieto, Patricio Pérez

**Affiliations:** 1 University of Cantabria Department of Economics Santander Spain; 2 Marqués de Valdecilla Research Institute (IDIVAL) Health Economics Research Group Santander Spain; 3 Universidad de Cantabria y Banco Santander Santander Financial Institute (SANFI) Santander Spain

**Keywords:** Healthcare, Unmet needs, Spain, Discrete choice models, Employment, Atención sanitaria, Necesidades no cubiertas, España, Modelos de elección discreta, Empleo

## Abstract

**Background:**

This study examines the relationship between unmet healthcare needs and employment status, with a focus on how this relationship was influenced by the Spanish economic recession of 2008-2012.

**Methods:**

Data were obtained from the Spanish National Health Survey for 2006 and 2011-2012. The outcome variable was the presence of unmet healthcare needs, describing its reasons. The analysis included the period (pre-crisis/crisis), demographic variables (sex, age, Spanish citizenship), employment status, social factors (level of education, marital status, social-class), and health indicators (self-assessed health, chronic conditions, and limitations). Logistic regression was used to predict unmet health needs based on the period, employment status, and control variables.

**Results:**

The frequency of unmet healthcare needs was low and decreased further during the crisis (5% pre-crisis *vs.* 3% during the crisis). Unmet healthcare needs were more strongly associated with health status than with employment status. However, among the unemployed, unmet healthcare needs increased during the crisis compared to the pre-crisis.

**Conclusions:**

The most vulnerable groups, characterized by higher unmet healthcare needs, included women, individuals with lower levels of education, and those in poorer health. These groups may require more targeted attention. These findings should be interpreted in the context of the Spanish National Health System, which is fully decentralized and provides healthcare and protection to all residents.

## RESUMEN

**Fundamento:**

Se analiza la relación entre las necesidades de atención médica no cubiertas (UHN) y la situación laboral, y si esta relación se vio modificada por la recesión económica de 2008-2012.

**Metodología:**

Se obtuvieron datos de la Encuesta Nacional de Salud de España de 2006 y de 2011-2012. La variable de resultado fue la existencia de UHN, describiéndose sus motivos. Se consideró el periodo (pre-crisis/crisis) y variables demográficas (sexo, edad, ciudadanía española), de empleo, sociales (nivel de estudios, estado civil, clase social) y de salud (autoevaluada, cronicidad y limitaciones). Mediante regresión logística se predijo la existencia de UHN según periodo, situación laboral y variables de control.

**Resultados:**

La frecuencia de UHN fue baja y disminuyó aún más durante la crisis (5 *vs.* 3%). Las UHN se asociaron más con el estado de salud que con la situación laboral. En todo caso, las necesidades de atención médica no cubiertas de los desempleados aumentarían en el período de crisis en comparación con el período anterior a la crisis.

**Conclusiones:**

Los grupos más vulnerables (con más necesidades de atención médica no cubiertas) fueron las mujeres, las personas con menos nivel educativo, y los individuos menos sanos, por lo que requerirían una atención más pormenorizada. Estos hallazgos deben interpretarse considerando que el Sistema Nacional de Salud español, completamente descentralizado, brinda atención médica y protección a todos los residentes.

## INTRODUCTION

A growing consensus in the literature suggests that inequality within the population can hinder growth by depriving the poor of the ability to maintain good health and accumulate human capital^1^. Therefore, health protection has become a central objective of health policies in modern welfare states. Health protection encompass a range of activities, but this study focuses specifically on access to healthcare. Various tools exist to monitor healthcare accessibility and assess inequities in access and service utilization, with unmet healthcare needs (UHN) being one of the most important and straightforward indicators^2^.

UHN contribute to health inequalities and disparities in healthcare access. When these needs disproportionally affect vulnerable groups, they exacerbate existing inequities. As a result, UHN are commonly used as an intermediate indicator to evaluate healthcare systems. Indeed, the most widely used indicator in the literature for assessing access (or lack thereof) to healthcare is UHN^3^^,^^4^. In this study, we test the hypothesis that UHN (self-reported UHN in the past 12 months) can be understood in terms of both individuals’ socioeconomic characteristics (ISC) and economic conditions, particularly during times of crisis. Special attention is given to employment status, as it is associated with both health and various ISC. The Spanish healthcare system and available data make UHN our main and sole variable, despite being a self-reported measure.

Employment status raises the question of whether unemployed individuals report more or fewer UHN. Is there an economic motivation behind this? A clear relationship exists between employment status, income, and health^5^. The negative effect of unemployment on health have been explained by various theories^6^. Moreover, UHN can lead to worse health outcomes, which in turn can increase unemployment. Unemployment primary results in the loss of income, which deteriorates overall well-being. Additionally, unemployed individuals may face barriers to healthcare access, such as waiting lists, as they are less likely to afford private healthcare services^7^. Time availability could also play a significant role^8^. In a system like Spain’s, with universal public health, employed individuals might be less likely to seek necessary healthcare due time constraints compared to the unemployed. Madureira-Lima et al.^9^ examined whether unemployment increased the risk of UHN during the Great Recession across Europe and found that job loss indeed raised the risk. Furthermore, lower out-of-pocket health expenses or higher income replacement for the unemployed helped protect against loss of healthcare access, but did not guarantee it. The authors concluded that policy actions should secure financial protection for the unemployed to prevent them from being concerned about their health.

Why this topic? And why Spain? Spain provides a unique context due to the significant rise in unemployment rates following the 2008 financial crisis^10^^,^^11^. According to Eurostat, the unemployment rate in Spain rose from 8.2% in 2007 to 26.1% in 2013, while the unemployment rate for the European Union-28 increased from 7.2% and 10.9% during the same period. In response, the Spanish Government implemented Royal Decree Law 16/2012, the most significant health system reform in recent years, aimed at controlling health expenditures and ensuring the sustainability of the National Health System. Notably, Spain’s health protection system evolved from a Social Security model to a National Health System (NHS) with the 1986 Health Act. Ensuring universal coverage to healthcare and regional decentralization. However, the Royal Decree-Law 16/2012 reduced universal coverage and introduced co-payments for prescription drugs.

Thus, the goal of this study is to examine two main hypothesis: i) UHN are associated with employment status and ii) this relationship is influenced by the economic cycle. To explore these hypotheses, we compare the pre-crisis and crisis periods specifically focusing on the Great Recession.

## MATERIALS AND METHODS

The analysis required individual-level data on the characteristics of the adults, as well as a source of time variation (considering the periods before and during the Great Recession). Microdata were obtained from the Spanish National Health Survey (SNHS), a research initiative conducted by the Spanish National Institute of Statistics (INE) in partnership with the Ministry of Health. Similar to other European health surveys, the SNHS provides national and regional data on various health-related issues. However, unlike a panel survey, it does track the same sample of households over multiple years. We used the 2006 and 2011-2012 surveys, as they were available and comparable for the periods just before and during the 2008 credit crunch (data from the intervening years, 2007-2010, as well as from 2013-2016 were unavailable). We limited the sample to the working-age population (ages 16-65) as our primary focus was on the effect of the employment status.

The main dependent variable was UHN, defined as a binary variable^12^, which takes 1 = affirmative response to the question: *Was there any time during the last 12 months when, in your opinion, you needed a medical examination or treatment but you did not receive it?* Respondents who answered affirmatively were then asked to indicate the main reason they did not see a doctor when needed. For descriptive purposes, we grouped the reasons with the greatest possible caution, as the two surveys included different items.

The variable *time* represented the period in which the individual was assessed: crisis or pre-crisis: t = 1 for the period 2011-2012 (crisis), and t = 0 for 2006 (pre-crisis).

The following individual characteristics were recorded:

- Demographic: gender (female, male), age (years), citizenship (Spanish, other).
- Employment status: employed, unemployed, inactive (student, housework, or retired).
- Social: education level (primary, secondary, pre-university, university, in training), marital status (single, married, separated/divorced, widowed), social class (based on the occupation of the reference person: 1. directors and managers of establishments with ≥ 10 employees and professionals traditionally associated with university degrees; 2. directors and managers of establishments with < 10 employees and professionals traditionally associated with university diplomas, as well as other technical support professionals, athletes, and artists; 3. intermediate occupations and self-employed workers; 4. supervisors and workers in qualified technical occupations; 5. qualified workers from the primary sector and other semi-skilled workers; 6. unskilled workers.
- Health: self-assessed health (very good, good, fair, bad, or very bad), health conditions (chronic: suffering from any chronic condition; limitations: if having any restricts in daily activities).

## Statistical analysis

Age is presented as the mean, while other variables are presented as relative frequency (proportions or percentages). Variables were not compared between the two periods, as only one data point (per variable) was available for each period.

Relationships between all studied variables and UHN were examined by grouping the variables (demographic, labor, social, and health), both with and without adjustment for the period (*time*), and globally, resulting in a full model adjusted for all variables. Relationships between the binary dependent variable UHN and the employment status and time were analyzed using a multivariate binary logistic regression model. This model included the parameters to be estimated (*odds ratio*, OR), the vector of control variables (*X*), unemployment status (UNEM: 1=yes/0=no), the survey year (*t*: 1=crisis/0=pre-crisis), and *ε*, the error term:

We used UNEM to test hypothesis i *(UHN are associated with employment status*) and its interaction with the period (UNEM*t) to test hypothesis ii (*the relationship between UHN and employment status is modified by the economic cycle, comparing the periods before, and during the Great Recession*). Thus, we captured the effects of being unemployed (UNEM) regardless of the period, being in the crisis period (t), and/or being employed during the crisis period (UNEM*t).

## RESULTS

The samples consisted of 31,300 and 24,000 dwellings, distributed across 2,236 and 2,000 census sections for 2006 and 2011-2012, respectively. After restricting the sample to the working-age population (16-65 years), final sample sizes were 21,537 for 2006 and 14,912 for 2011-2012.

Table 1 presents the characteristics of the sample for both periods (pre-crisis and crisis). The results were generally similar between periods, despite the fact that SNHS is not a panel survey where the same households were interviewed year after year. Specifically, slightly over half of the sample were female, with a mean age of 42 years, and most were employed and married. During the crisis period, compared to the pre-crisis period, the proportion of unemployed individuals doubled from 0.08 to 0.17. Additionally, the proportion of individuals with secondary education increased from 0.14 to 0.35, while those with primary education decreased from 0.36 to 0.13. Social class 5 increased from 0.20 to 0.31, while social class 4 decreased from 0.21 to 0.15. Self-assessed health showed a decrease in those reporting *very good* health and an increase in those reporting *fair* health. The proportion of individuals with chronic diseases rose from 0.23 to 0.38, while the proportion reporting limitations decreased from and 0.21 to 0.16.

**Table 1:** Characteristics of the studied variables for both samples (proportions)

Variable	Pre-crisis -2006	Crisis (2011-2012)
*Demographic*		
Gender (female)	0.57	0.51
Age (years), mean	42.14	42.84
Citizenship (Spanish)	0.91	0.92
*Employment status*		
employed	0.60	0.57
unemployed	0.08	0.17
inactive	0.32	0.26
*Social*		
Education level		
primary	0.36	0.13
secondary	0.14	0.35
pre-university	0.23	0.25
university	0.19	0.19
labor training	0.08	0.08
Marital status		
single	0.31	0.35
married	0.60	0.54
separated/divorced	0.06	0.08
widowed	0.03	0.03
*Social class*		
1	0.08	0.12
2	0.10	0.08
3	0.24	0.20
4	0.21	0.15
5	0.20	0.31
6	0.17	0.14
*Health*		
Self-assessed health		
very good	0.17	0.22
good	0.54	0.55
fair	0.22	0.17
bad or very bad	0.07	0.06
Health conditions		
chronic	0.23	0.38
limited	0.21	0.16

UHN were already low in 2006 (only 5.10%) and even lower during the crisis (2.68%). Regarding the reasons for UHN, *financial motives* were infrequent in 2006 and further decreased during the crisis period. *Other reasons* accounted for nearly half of the reported UHN in 2006, but decreased by 20 percentage points in 2011-2012. During this later period, a new reason *-wait-and-see-*, was introduced, becoming the third most frequent reason for UHN during the crisis. The *waiting list* was the second most common reason in 2006, but became the most frequent in 2011-2012 (Table 2).

**Table 2:** Percentages of unmet health needs and its main reasons

Reason	Pre-crisis -2006	Crisis (2011-2012)
Financial reasons	5.59	4.51
Waiting list	32.88	39.35
Lack of time	8.39	8.52
Distance, transport difficulties	1.45	0.75
Fear	2.51	1.25
*Wait-and-see* attitude	-	16.54
Other reasons	49.18	29.07
Total UHN	(n = 1,037)	(n = 399)
	5.10	2.68

When first considering the group of variables (crude), including demographic, labor, social, and health, we found that female subjects, non-Spanish citizens, individuals who were separated or divorced, and those in poorer health were more likely to report UHN. Conversely, a lower probability of reporting UHN was observed for individuals in the crisis period, those with primary or pre-university education, and those in social classes 1 and 2. Regarding employment status, the unemployed appeared to have a higher likelihood of reporting UHN.

These results remained largely consistent for demographic and health factors when adjusted for the period. However, discrepancies were seen for social and labor factors. Specifically, after adjusting for the period, we found that when the group of variables (demographic, labor, social, and health) adjusted by period (*time*) were related to UHN, we found that females, non-Spanish citizens, individuals who were separated or divorced, separated/divorced, and those in poorer health continued to report UHN more frequently. Regarding social variables, only marginally significant effects were observed for social class. Employment status, however, showed no significant association with UHN after adjustment.

In the full model, the variables significantly associated with UHN were time (pre-crisis period), being a female, not having a Spanish citizenship, being in labor training, being separated or divorced, self-assessed health less than *very good*, and having chronic conditions and/or limitations in daily life activities (Table 3).

**Table 3:** Relationship between variables and unmet healthcare needs (logistic regression)

	Multivariate logistic regression model OR (95% CI)		
Variable	By groups of variables		Full model
	Crude	Adjusted by time	
Time	0.54 (0.48-0.61)		0.49 (0.42-0.56)
*Demographic*			
Gender (female)	1.59 (1.42-1.78)	1.53 (1.36-1.70)	1.43 (1.27-1.61)
Age	1.00 (1.00-1.00)	1.00 (1.00-1.00)	0.99 (0.98-1.00)
Citizenship (Spanish)	0.73 [0.62-0.87]	0.74 (0.62-0.87)	0.71 (0.59-0.85)
*Employment status*			
employed	reference	reference	reference
unemployed	1.51 (1.22-1.87)	1.20 (0.97-1.50)	0.93 (0.75-1.17)
unemployed*t	0.65 (0.49-0.87)	1.29 (0.94-1.77)	1.38 (1.01-1.91)
inactive	1.06 (0.94-1.19)	1.03 (0.92-1.17)	0.75 (0.66-0.86)
*Social*			
Education level			
primary	0.84 (0.71-1.00)	0.93 (0.77-1.11)	0.85 (0.70-1.03)
secondary	0.96 (0.83-1.11)	1.15 (0.99-1.35)	1.11 (0.94-1.30)
pre-university	0.82 (0.71-0.94)	1.10 (0.94-1.28)	1.09 (0.94-1.28)
university	reference	reference	reference
labor training	1.14 (0.93-1.41)	1.37 (1.10-1.70)	1.37 (1.10-1.70)
Marital status			
single	reference	reference	reference
married	1.02 (0.90-1.15)	0.99 (0.88-1.12)	0.95 (0.83-1.08)
separated/divorced	1.57 (1.30-1.91)	1.57 (1.30-1.91)	1.27 (1.03-1.56)
widowed	1.15 (0.85-1.56)	1.15 (0.85-1.56)	0.97 (0.70-1.35)
Social class			
1	0.58 (0.45-0.75)	0.74 (0.57-0.96)	0.95 (0.73-1.24)
2	0.77 (0.61-0.97)	0.87 (0.69-1.09)	0.96 (0.73-1.24)
3	0.90 (0.76-1.07)	0.95 (0.80-1.12)	1.05 (0.88-1.25)
4	0.98 (0.83-1.16)	1.00 (0.84-1.18)	1.12 (0.95-1.35)
5	0.95 (0.81-1.11)	1.05 (0.89-1.22)	1.03 (0.87-1.21)
6	reference	reference	reference
*Health*			
Self-assessed health			
very good	reference	reference	reference
good	1.58 (1.30-1.92)	1.52 (1.25-1.85)	1.60 (1.31-1.95)
fair	2.36 (1.90-2.93)	2.13 (1.72-2.65)	2.37 (1.90-2.96)
bad or very bad	2.88 (2.23-3.70)	2.56 (1.99-3.30)	3.10 (2.39-4.02)
Health conditions			
chronic	1.29 (1.14-1.47)	1.50 (1.31-1.72)	1.57 (1.37-1.80)
limit	2.13 (1.86-2.45)	1.97 (1.72-2.28)	2.03 (1.77-2.34)
*Constant*			0.03 (0.03-0.05)
*Observations*	34,449	34,449	36,402

OR: *odds ratio*; CI: confidence interval. 1: reference category. In bold, significant effects (p <0.05).

## DISCUSSION

In recent years, there has been an increase of empirical studies using new data to analyze the demand for healthcare services in general, and issues related to UHN in particular. However, there is limited updated literature on UHN specifically for Spain. This work, focuses on the impact of austerity on healthcare and includes recent works focused on UHN, both from international^12^^-^^19^ and Spanish^20^^-^^23^ perspectives.

We present an empirical approach to address this gap in the literature. Several features distinguish this study from previously published research. First, to the best of our knowledge, it is one of the few studies that examines UHN using data from the Spanish National Health Survey. Second, the study differentiates between distinct stages of the economic cycle, specifically comparing 2006 and 2011-2012. In summary, the originality of this work lies in its empirical analysis of UHN in Spain across two different economic periods. Thus, the study stems from its combination of theoretical and empirical perspectives.

Economic downturns and recession often lead to budget cuts and service reductions in the healthcare sector, which can precipitate shifts in the distribution of healthcare resources^24^. From a policy perspective, ensuring equity of access to healthcare services is a key objective. We believe that this contribution will be valuable for policymakers as they work to improve health outcomes^25^^-^^28^.

As expected, our empirical results align with previous international studies, which suggests that certain population groups are more vulnerable to experiencing UHN, with a higher probability of reporting UHN^13^^-^^17^^,^^20^^-^^22^^,^^29^^,^^30^. These vulnerable groups include women and individuals with lower education levels or poorer health. Policy implications for healthcare should focus on these groups to ensure more equitable access to services.

These findings warrant further investigation, and the lack of detailed information regarding the *reasons for reporting UHN* is particularly important^13^. Our results for Spain, both prior to and during the Great Recession, suggest that the worsening of healthcare access during the crisis was not as pronounced as expected. Despite the anticipated increase in barriers to healthcare access in Spain due to Decree Law 16/2012, the overall prevalence of UHN actually decreased from 5% to 3%. However, it is important to note that the respondents were not the same in each period (2006 *vs*. 2011-12), and the most affected -immigrants- was not statistically representative in this sample.

The strongest association with UHN was found for health-related variables. In contrast, UHN showed a weaker relationship with employment status, which may be largely due to a selection effect, as UHN is primarily health-related. Hypothesis i (UHN are associated with employment status) was rejected, while hypothesis ii (the economic cycle modifies the relationship between UHN and employment status) was supported. Specifically, the prevalence of UHN among unemployed individuals increased during the crisis period compared to the pre-crisis period.

Nonetheless, our results must be interpreted with caution, as other contextual factors (such as policies, characteristics of the services, and the National Healthcare System, etc.) and structural factors (e.g., the care provided) may have influenced UHN across the two study periods. Specifically, our findings should be considered in the context of the Spanish National Health Service, which provides *universal coverage*. Additionally, it should be noted that efforts to maintain healthcare service standards would likely play a significant role in this framework^31^.

Furthermore, it is important to acknowledge the limitations and potential extensions of the available data. Although the SNHS provides valuable individual-specific data, one limitation is the subjectivity of the responses, as they are based on individual perceptions and expectations. This subjectivity increases the likelihood of recall bias, although no more objective data could be obtained from the available microdata in these surveys. Additionally, the respondents in 2006 were not the same as those in 2011-2012, which may also affect the comparability of the data. Moreover, analyzing the differences in the primary reason for UHN was not possible with the available data. Rather than diminishing the significance of these limitations, they should encourage further research on an issue of critical importance to Spain’s citizens. Health inequality is an area that requires greater effort to generate empirical data for policymakers, especially as it may be influenced by the decentralization process across Spain’s regions and their political dynamics.

This study could be expanded in several ways. For instance, it would be valuable to test our findings using alternative econometric methods and control variables or to conduct a multiple-country study. These and other extensions of this research are left for further research.

In conclusion, the frequency of UHN decreased during the crisis period. UHN was more prevalent among women, individuals with chronic diseases, those with limitations, and individuals who rated their health as *less than very good*. Conversely, UHN were less common among people with Spanish citizenship and those who were inactive in the labor market. These findings should be interpreted within the context of the Spanish National Health System, which is fully decentralized and provides medical care and protection to all residents.

## Data Availability

Data are available under request to corresponding author.
